# Book Review: Understanding young onset dementia: Evaluation, needs and care

**DOI:** 10.1177/14713012231151988

**Published:** 2023-01-16

**Authors:** Mariko Sakamoto

**Affiliations:** 8166 University of British Columbia, Vancouver, BC, Canada

Dementia in its many forms affects millions of people worldwide, and is most often understood as a condition that affects older individuals. However, Young Onset Dementia (YOD), most often understood as diagnosis before age of 65, can affect up to 9% of those living with dementia (WHO, 2017). Drs. Marjolein de Vugt and Janet Carter’s edited book *Understanding young onset dementia: Evaluation, needs and care* (2021) addresses many of the specific challenges and needs of younger people living with dementia. Written for scholars, academics and students interested in the fields of mental health and dementia, this book is a collection of chapters written by international researchers and experts in the field. As its title implies, the book focuses on evaluation of YOD, and the particular needs and care considerations of those living with the condition. It takes a prosaic approach to fulfilling these objectives, organizing its contents into four parts. Part I focuses on nomenclature and clinical definitions, along with the epidemiology and subtypes of YOD. Part II includes a focus on clinical presentations of dementia in younger people, with a chapter devoted to behavioural and emotional challenges. Part III moves into the realm of lived experience, and details both care and service needs, as well as the perspectives of the children of those who live with the condition. Part IV focuses more specifically on care and support, including an overview of international best practices and end of life considerations.

The first sections of the book (Part I & II), provide a descriptive account of the heterogeneity of YOD, in terms of the many ways the condition can present itself in younger individuals. In addition, the challenges that many with YOD experience, when trying to receive a timely and accurate diagnosis, are discussed. These sections of the book are focused largely on epidemiology and clinical presentations, and would likely be of more interest to clinicians typically involved in diagnosis and treatment, or scholars who study the epidemiology of dementia. The last chapter (Chapter 4) in Part II focuses on changes in behaviour and emotions, using the term neuropsychiatric symptoms to encapsulate these challenges. The focus of this chapter is quite clinical, detailing the behaviours and emotional changes people can experience, and includes discussion on how behaviours can be managed. Chapter 4 does attempt to frame these difficulties within understandings of unmet needs, pointing out that person-centered and psychosocial interventions are the preferred approach. Nevertheless, the reader is left wanting a more fulsome discussion on this sensitive topic, especially as there is a tendency in care settings and in the literature for dementia-related behaviours to be pathologized without full consideration of factors, such as unmet needs, that contribute to how people with dementia express themselves emotionally and behaviourally (Dupuis et al., 2012).

The book’s strengths lie in its second half when focus shifts to the lived experience of YOD and where important topics such as relationship changes and challenges, vocational and work-related concerns, and end of life needs are explored. The use of the term “off time condition” (p. 67) in Chapter 5 is apt when considering that dementia is most often diagnosed in older people and that a diagnosis of YOD can disrupt the middle years of a person’s life. For those seeking better understanding of YOD, this section of the book provides a helpful account of what it is like to experience symptoms, seek help and receive a diagnosis. Attention is poignantly drawn to the impact of YOD on people’s sense of identity, as well as the grief and loss experienced by the children of people with the condition, who are often youths or young people themselves. A common theme throughout the discussion in many of the chapters is the lack of and need for research in the field of YOD. As such, the chapters in the latter half of the book, which summarize and lay out existing gaps in knowledge, would be of use to scholars interested in YOD research and addressing care and service needs. Throughout the book, it is made clear that people with YOD are a distinct population, and that person-centered supports and resources are needed that are tailored to them as younger people experiencing dementia. Fortunately, Chapter 8 conveniently lays out an overview of international programs as examples of best practice and guidelines that would be useful for policy and decision makers.

While the intention of this book may have been to emphasize the specific needs of younger individuals living with dementia, it is notable that there is little discussion of the stigma that is a significant aspect of living with the condition (Swaffer, 2014). This topic is touched upon – for instance, stigma is mentioned in the discussion of the diagnosis’ impact on younger people’s sense of identity. There is further mention of the social stigma associated with a dementia diagnosis, in particular for family members and carers of those living with the condition. Given that this book highlights that many existing services and resources are designed for older people living with dementia, it is surprising that stigma is not discussed more fully, in terms of the stigma that younger people can experience as people dealing with a condition most often associated with later life. This is a gap in this book, as stigma and discrimination are powerful forces that can affect how services are provided, resources are allocated, and research is undertaken, with significant impact on the day-to-day experiences of people living with YOD. Addressing dementia-related stigma is a priority for people with dementia (Bethell et al., 2018), regardless of age. A such, future editions would benefit from exploring this topic more fully. Nevertheless, as a collection of materials from various experts, this book provides a broad overview of the research contributions in the field of YOD and is a good place to start for clinicians, care providers and researchers interested in learning more about diagnosing, treating, and supporting people living with this condition.

## ORCID iD

Mariko Sakamoto https://orcid.org/0000-0002-1164-0324
